# The effectiveness of postacute intensive rehabilitation on severe COVID‐19 patients: A case‐control study

**DOI:** 10.1002/hsr2.1506

**Published:** 2023-08-18

**Authors:** Márlon J. R. Aliberti, Marcelo R. Levites, Frederico A. N. Berardo

**Affiliations:** ^1^ Laboratorio de Investigacao Medica em Envelhecimento (LIM‐66) Serviço de Geriatria, Hospital das Clinicas HCFMUSP, Faculdade de Medicina da Universidade de Sao Paulo Sao Paulo Brazil; ^2^ Research Institute Hospital Sirio‐Libanes Sao Paulo Brazil; ^3^ Department of Internal Medicine Post‐Acute Care Premium Care Sao Paulo Brazil

**Keywords:** COVID‐19, functional status, postacute COVID‐19 syndrome, rehabilitation, subacute care, transitional care

## 1 INTRODUCTION

Coronavirus Disease 2019 (COVID‐19) can cause multiorgan failure and longer‐term consequences, particularly in patients requiring in‐hospital care.^1^, ^2^, ^3^, ^4^ Studies indicate that approximately 60% of these patients have persistent symptoms—commonly called “long COVID”—such as fatigue, breathlessness, and joint pain.^5^, ^6^, ^7^ These long‐term effects hinder a smooth transition back to independent living, which encompasses challenges for performing essential self‐care activities.^2^ Although similar manifestations have been documented in patients recovering from other critical illnesses, emerging evidence suggests that severe COVID‐19 patients may encounter distinctive challenges. For instance, earlier research reported a higher rate of muscle mass and strength reduction in COVID‐19 patients than in other acutely ill individuals.^8^ Such findings imply a unique recovery trajectory for COVID‐19 patients, diverging from those of other critically ill patients.^2^, ^7^

Evidence shows that postacute intensive rehabilitation can help these patients restore function, avoid hospital readmissions, and improve satisfaction with received care.^3^, ^4^, ^5^ Despite such potential benefits, the characteristics of postacute care in patients who have experienced COVID‐19 compared to those with other well‐known respiratory infections, such as pneumonia caused by influenza or bacterial pathogens, remain unclear.^4^, ^5^, ^6^ This is a relevant issue as post‐COVID‐19 conditions become widespread, with millions of people recovering from the disease as the pandemic advances.^6^, ^7^, ^8^ Therefore, we conducted a case‐control study comparing clinical and functional outcomes of patients admitted to intensive postacute rehabilitation for COVID‐19 and other non‐COVID pneumonia.

## 2 METHODS

A case‐control study using data from a large postacute care institution in Sao Paulo, Brazil. This institution comprises seven facilities with a total of 243 beds and serves patients from over 40 different acute hospitals.^9^ Once patients have stabilized from a critical event, they are transferred from the acute hospital to a postacute facility for multidisciplinary care to restore function, avoid readmissions, and facilitate the transition to the community.^9^

A multidisciplinary team of physicians, nurses, therapists (physical, occupational, and speech‐language), nutritionists, social workers, pharmacists, and psychologists collaboratively deliver intensive rehabilitation services (Supporting Information: Table S1). With round‐the‐clock health monitoring, prompt intervention and nursing care are ensured. Individualized treatment plans incorporate at least two rehabilitation modalities, providing 3 h of daily therapy for holistic recovery that encompasses physical, cognitive, and psychosocial abilities as needed.^9^

We assessed data from patients aged ≥ 8 years consecutively admitted to the seven postacute facilities between June 2020 and June 2022. For this study, we considered eligible patients admitted for intensive rehabilitation due to COVID‐19 (cases) and those due to other pneumonia (controls). We excluded readmissions of patients previously included in analyses and those with missing data.

Trained physicians collected data from electronic health records filled out by healthcare professionals during patient admission and discharge. They registered information on sociodemographic factors, Charlson comorbidity index, physiological measurements, infectious agents, dependence in basic activities of daily living (ADL) according to the Katz index (0−6), presence of pressure ulcers, and need for supplemental oxygen, enteral nutrition, and aspiration. They also documented important outcomes at the end of postacute care admission, including length of stay, discharge destination or death, and satisfaction levels.

The Research Ethics Committee of University of Sao Paulo approved the study (approval number 5.153.577) and waived the need for obtaining written consent.

For the analysis, first, we matched cases (COVID‐19) and controls (other pneumonia) based on sociodemographic factors (age, sex, race/ethnicity, civil status, living arrangements, education), Charlson comorbidity index, and any ADL dependence on postacute care admission. Second, we reported categorical variables as count and percentage and interval variables as median and interquartile range (IQR) or mean and standard deviation (SD). Third, we compared characteristics and outcomes between cases and controls using the Fisher exact test for categorical variables and Mann−Whitney test or independent samples *t*‐test for the interval variables. Fourth, we examined differences in clinical characteristics at discharge versus admission within each group using the McNemar's test. Finally, we used linear mixed models to investigate the impact of intensive rehabilitation on the ADL function of cases (COVID‐19) versus controls (other pneumonia) in unadjusted and adjusted models. We incorporated as confounders into the multivariable model the following variables that could interfere in improving physical function in the context of patients recovering from infections: age, sex, race/ethnicity, civil status, living arrangements, education, Charlson comorbidity index, physiological measurements, year of admission, presence of pressure ulcers, need for aspiration and enteral nutrition, and length of stay.

All tests were two‐tailed, with statistical significance set as <0.05. Analyses were performed using Stata version 17.0 (StataCorp).

## 3 RESULTS

Out of 603 intensive rehabilitation admissions, we initially selected 162 patients for our study (107 with COVID‐19 and 55 with other types of pneumonia). This selection excluded 417 admissions not due to pneumonia, 12 with missing data, and 12 readmissions. After applying matching procedures, we obtained a final sample of 150 patients: 100 with COVID‐19 and 50 with other types of pneumonia (Supporting Information: Figure S1). The most common infectious agents for other pneumonia in the control group were bacteria (36%), influenza (22%), adenovirus (8%), and unknown (34%). Sociodemographic factors, comorbidities, and ADL dependence did not vary between cases and controls on admission (Table 1). We observed clinically significant improvements in the need for supplemental oxygen, aspiration, enteral nutrition, and ADL dependence at discharge compared to admission in those admitted for COVID‐19 (Supporting Information: Table S1).

**Table 1:** Characteristics and outcomes of patients admitted to postacute intensive rehabilitation according to COVID‐19 status.

Variables^a^	Total	COVID‐19	Control^b^	*p* Value^c^
	(*n* = 150)	(*n* = 100)	(*n* = 50)	
*Characteristics on admission*				
Age (years), mean (SD)	64.8 (15.8)	64.5 (13.9)	65.4 (19.3)	0.74
Female sex, *n* (%)	71 (47.3)	45 (45.0)	26 (52.0)	0.42
White race/ethnicity, *n* (%)	117 (78.0)	77 (77.0)	40 (80.0)	0.91
Married, *n* (%)	76 (50.7)	51 (51.0)	25 (50.0)	0.72
Living with family, *n* (%)	137 (91.3)	92 (92.0)	45 (90.0)	0.66
Education (years), median (IQR)	11 (10−14)	11 (9−14)	11 (10−12)	0.50
Charlson comorbidity index, median (IQR)	2 (1−3)	2 (1−2)	2 (1−3)	0.89
National Early Warning Score (NEWS), median (IQR)^d^	3 (2−5)	3 (2−5)	3 (0−5)	0.81
Supplemental oxygen, *n* (%)	87 (58.0)	68 (68.0)	19 (38.0)	<0.001
Airway suctioning, *n* (%)	35 (23.3)	22 (22.0)	13 (26.0)	0.59
Enteral nutrition, *n* (%)	44 (29.3)	28 (28.0)	16 (32.0)	0.61
Pressure ulcer, *n* (%)	17 (11.3)	12 (12.0)	5 (10.0)	0.72
Dependence in performing ADL, *n* (%)^e^	133 (88.7)	89 (89.0)	44 (88.0)	0.86
*Rehabilitation therapies*				
Physical therapy	144 (96.0)	96 (96.0)	48 (96.0)	1.00
Occupational therapy	105 (70.0)	69 (69.0)	36 (72.0)	0.71
Speech therapy	118 (79.0)	75 (75.0)	43 (86.0)	0.12
Nutritional therapy	144 (96.0)	96 (96.0)	48 (96.0)	1.00
*Outcomes at discharge*				
Length of stay (days), median (IQR)	33.5 (13−76)	30 (9−54.5)	78.5 (23−90)	<0.001
Discharge destination, *n* (%)				0.45
Home	124 (82.7)	84 (84.0)	40 (80.0)	
Hospital readmission	23 (15.3)	15 (15.0)	8 (16.0)	
Death	3 (2.0)	1 (1.0)	2 (4.0)	
Dependence in performing ADL, *n* (%)	74 (49.3)	39 (39.0)	35 (70.0)	<0.001
Level of satisfaction (0–10), *n* (%)				0.80
Very dissatisfied to dissatisfied (0−6)	14 (9)	8 (8)	6 (12)	
Neutral (7−8)	5 (3)	4 (4)	1 (2)	
Satisfied to very satisfied (9−10)	131 (87)	88 (88)	43 (86)	

^a^ We reported categorical variables as count and percentage and interval variables as mean and standard deviation (SD) if normal‐distributed or median and interquartile range (IQR) if non‐normal distributed.

^b^ The control group comprised patients admitted to postacute intensive rehabilitation for other non‐COVID pneumonia.

^c^ We compared COVID‐19 and control groups using the Fisher exact test for categorical variables and independent samples *t*‐test (normal distribution) or Mann−Whitney test (non‐normal distribution) for the interval variables.

^d^ NEWS is a simple aggregate scoring system based on six physiological measurements: respiration rate, oxygen saturation systolic blood pressure, pulse rate, level of consciousness, and temperature.

^e^ Dependence in at least one basic activity of daily living (ADL) according to Katz index (transferring, eating, continence, dressing, toileting, and bathing).

**Figure 1:**
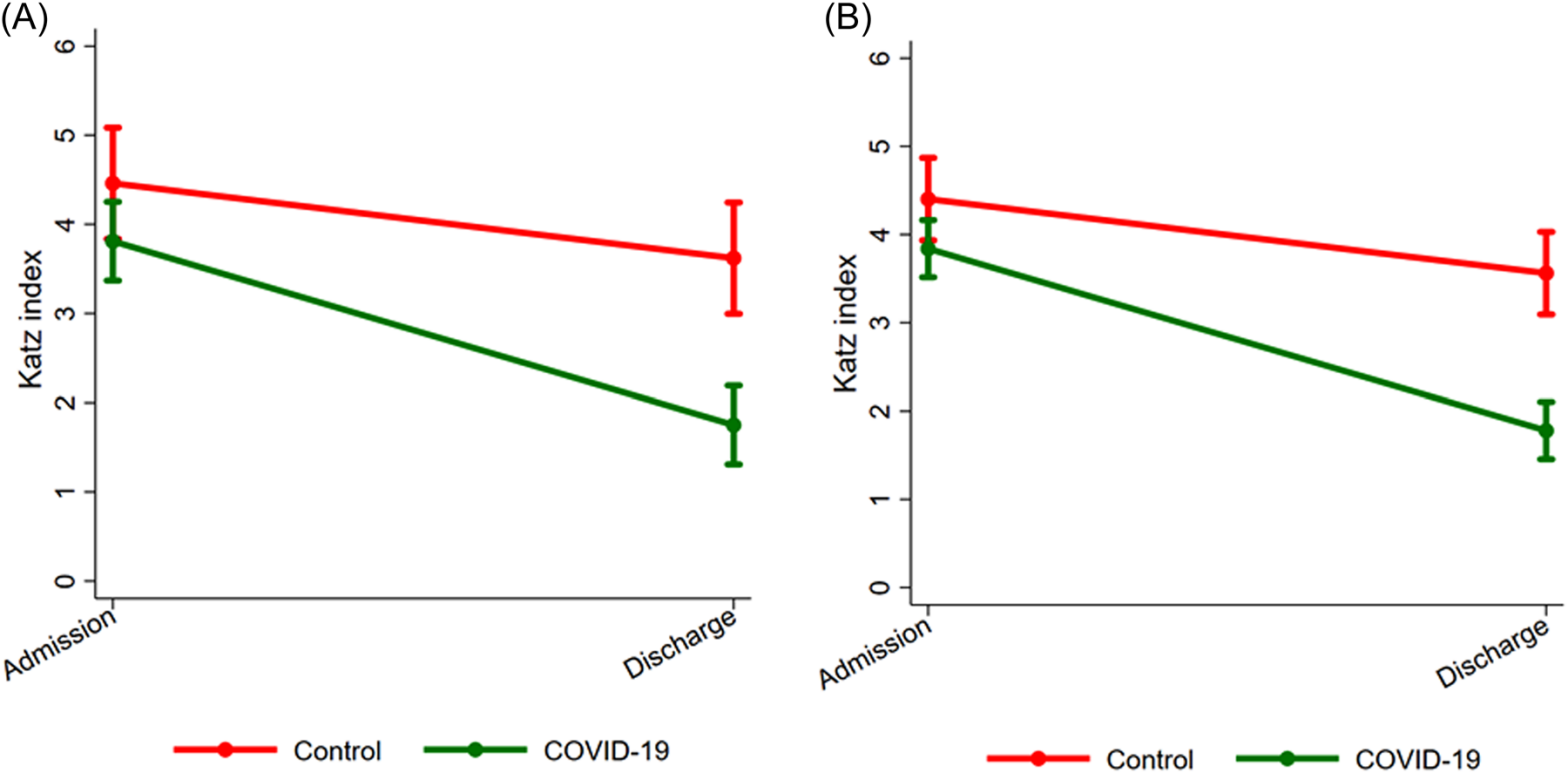
The impact of intensive rehabilitation on the ADL function of patients recovering from COVID‐19 and other pneumonia. (A) Unadjusted and (B) Adjusted for possible confounders. Point estimates and bars represent means and 95% confidence intervals for the Katz index (0−6; higher = worse), respectively. ADL = basic activities of daily living according to Katz index. We found that patients admitted for COVID‐19 had 1.2 times higher improvement (95% confidence interval = 0.5–2.0) in the Katz index at discharge compared to controls using unadjusted or adjusted linear mixed models. For this analysis, we used the Katz index (on admission and at discharge) as the outcome and the reason for admission (COVID‐19 vs. other pneumonia) as the primary variable of interest. In the adjusted analysis, we also incorporated confounders assessed on admission (age, sex, race/ethnicity, civil status, living arrangements, education, Charlson comorbidity index, physiological measurements, year of admission, presence of pressure ulcers, need for aspiration and enteral nutrition, Katz index) and at discharge (length of stay). ADL, activities of daily living.

Comparing the outcomes between cases and controls at the end of postacute care, we did not find differences in discharge destination or death and satisfaction levels; however, intensive rehabilitation for COVID‐19 was associated with shorter lengths of stay and less ADL disability (Table 1). In unadjusted and adjusted linear mixed models, patients with COVID‐19 had 1.2 times higher improvement (95% confidence interval = 1.3−2.7) in the Katz index at discharge than controls (Figure 1).

## 4 DISCUSSION

This case‐control study showed the positive impact of postacute intensive rehabilitation on COVID‐19 patients. Our results indicate that COVID‐19 patients had more improvements in their ADL function and spent shorter median stays in postacute care compared to patients with other non‐COVID pneumonia. There was also a remarkable reduction in the need for supplemental oxygen, aspiration, and enteral nutrition at discharge compared to admission in COVID‐19 patients. Interestingly, we observed high rates of survival, discharge to home, and satisfaction in cases and controls. These findings support previous work and advance our understanding of the effectiveness of postacute rehabilitation in the recovery of severe COVID‐19 patients.^4^, ^5^, ^6^

The study should be interpreted considering its limitations. First, our findings on intensive inpatient rehabilitation may not be generalizable to outpatient and at‐home rehabilitation models.^10^ Second, it is important to note that our control group was relatively small and consisted of patients with diverse infectious agents for pneumonia. The different types of pneumonia may vary in severity and treatment approaches, which might introduce confounding factors influencing the results. Finally, despite considering comprehensive patient information, we could not obtain detailed data on some variables (e.g., the level of function before SARS‐CoV‐2 infection, complications in acute hospitals and vaccination status), a common limitation in retrospective studies.

In conclusion, our study highlights the effectiveness of postacute intensive rehabilitation for severe COVID‐19 patients, as it shows improved outcomes compared to those with other non‐COVID pneumonia. To further advance our understanding in this area, future prospective studies should focus on estimating the impact of rehabilitation on postdischarge outcomes, especially in diverse settings.

## AUTHOR CONTRIBUTIONS

**Márlon J. R. Aliberti**: Conceptualization; data curation; formal analysis; funding acquisition; investigation; methodology; writing—original draft; writing—review and editing. **Marcelo R. Levites**: Conceptualization; data curation; writing—original draft; writing—review and editing. **Frederico A. N. Berardo**: Conceptualization; data curation; supervision; writing—original draft; writing—review and editing.

## CONFLICT OF INTEREST STATEMENT

The authors declare no conflict of interest.

## TRANSPARENCY STATEMENT

All authors have read and approved the final version of the manuscript. The lead author, Márlon J. R. Aliberti, had full access to all of the data in this study and takes complete responsibility for the integrity of the data and the accuracy of the data analysis. Dr. Aliberti also affirms that this manuscript is an honest, accurate, and transparent account of the study being reported; that no important aspects of the study have been omitted; and that any discrepancies from the study as planned (and if relevant, registered) have been explained.

## Supporting information

Supporting information.

Supporting information.

## Data Availability

The data that support the findings of this study are available from the corresponding author upon a reasonable request. The data are not publicly available due to privacy or ethical restrictions.
