# Supplementary material for: The effectiveness of postacute intensive rehabilitation on severe COVID‐19 patients: A case‐control study
Source: Health Sci Rep. 2023 Aug 18;6(8):e1506. doi: 10.1002/hsr2.1506 (PMC10439337; doi:10.1002/hsr2.1506)
Supplement: Supplementary file 1 — Supporting information. [file HSR2-6-e1506-s001.docx]

**The effectiveness of post-acute intensive rehabilitation on severe COVID-19 patients: a case-control study**

**Supplementary material**

This supplementary material for online only contains:

**Figure**

Supplementary Figure S1. Flowchart of the study participants

**Tables**

Supplementary Table S1. Multidisciplinary healthcare professionals and their roles in inpatient intensive rehabilitation post-acute care

Supplementary Table S2. Differences in clinical characteristics at discharge versus admission within each group: COVID-19 and other pneumonia


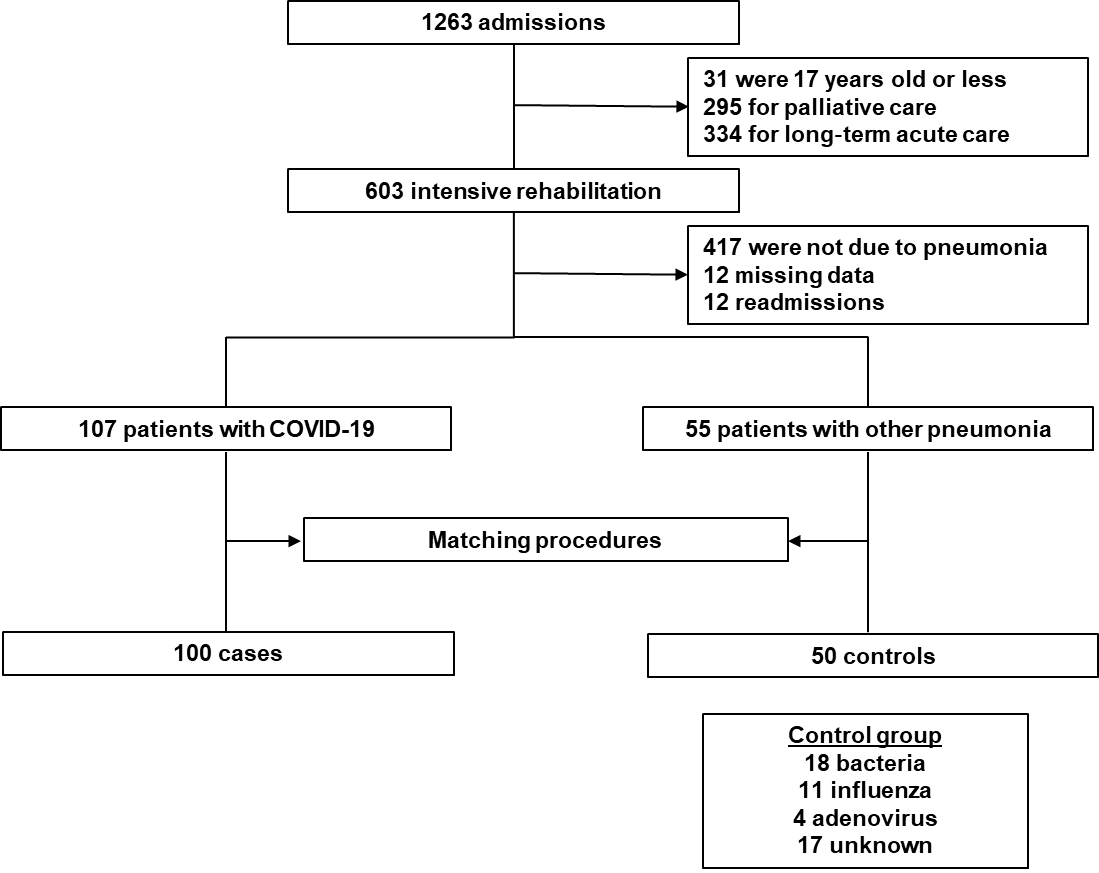


**Supplementary Figure S1.** Flowchart of the study participants

**Supplementary Table S1.** Multidisciplinary healthcare professionals and their roles in inpatient intensive rehabilitation post-acute care

| **Professional** | **Role and Activities** | **Frequency** |
| --- | --- | --- |
| **Physician** | As the team leader, the physician - preferably a physiatrist - conducts evaluations, provides medical management, and coordinates the overall care plan. A general practitioner also helps in clinical management, especially at night and on weekends. | Regular consultations as needed throughout the patient's stay and treatment progression. |
| **Registered**  **nurse** | The registered nurse provides specialized nursing care, administers medications, performs wound care management, conducts patient assessments, ensures coordination of patient care, and collaborates with other healthcare professionals. | Daily involvement in patient care. |
| **Nursing**  **assistant** | The nursing assistant supports the registered nurse in providing direct patient care, assists patients with activities of daily living, performs vital sign monitoring. | Daily involvement in patient care, working under the supervision and guidance of the registered nurse. |
| **Physical therapist** | The physical therapist focuses on improving mobility, strength, and functional abilities through tailored exercises and training sessions. | Daily therapy sessions, typically 2-3 times per day. |
| **Occupational therapist** | The occupational therapist assists patients in regaining daily living skills and promoting independence through therapy sessions and adaptive strategies. | As needed and scheduled therapy sessions, typically multiple times per week. |
| **Speech-language therapist** | The speech-language therapist addresses communication, swallowing, and cognitive impairments through therapy sessions and exercises. | As needed and scheduled therapy sessions, typically multiple times per week. |
| **Nutritionist** | The nutritionist assesses nutritional needs, develops individualized meal plans, and collaborates with the team to optimize nutritional intake. | Regular consultations throughout the patient's stay, based on nutritional assessment and needs. |
| **Social**  **worker** | The social worker assesses psychosocial needs, provides counseling, facilitates discharge planning, and coordinates community resources. | As needed and scheduled consultations based on the patient's psychosocial requirements. |
| **Pharmacist** | The pharmacist ensures appropriate medication management, reviews medication profiles, and provides recommendations for optimization. | Deliver pharmacological therapy daily and make regular consultations to review and optimize medication regimens. |
| **Psychologist** | The psychologist focuses on addressing the emotional and psychological aspects of recovery through counseling and support services. | As needed and scheduled consultations based on the patient's emotional and psychological needs. |

**Supplementary Table S2.** Differences in clinical characteristics at discharge versus admission within each group: COVID-19 and other pneumonia

|  | **COVID-19** | | ***P*-value^†^** | **Control** | | ***P*-value^†^** |
| --- | --- | --- | --- | --- | --- | --- |
| **Characteristics**, n (%) | **Admission** | **Discharge** |  | **Admission** | **Discharge** |  |
| **Supplemental oxygen** | 68 (68.0) | 17 (17.0) | <0.001 | 19 (38.0) | 9 (18.0) | 0.004 |
| **Airway suctioning** | 22 (22.0) | 5 (5.0) | <0.001 | 13 (26.0) | 5 (10.0) | 0.02 |
| **Enteral nutrition** | 28 (28.0) | 13 (13.0) | <0.001 | 16 (32.0) | 10 (20.0) | 0.06 |
| **Pressure ulcer** | 12 (12.0) | 6 (6.0) | 0.08 | 5 (10.0) | 3 (6.0) | 0.32 |
| **Dependence in performing ADL** ^‡^ | 89 (89.0) | 39 (39.0) | <0.001 | 44 (88.0) | 35 (70.0) | 0.003 |

^†^ We compared the patients’ characteristics at discharge versus admission within each group (COVID-19 and other pneumonia [control]) using the McNemar’s test. For the three patients who died, we considered information immediately before death.

^‡^ Dependence in at least one basic activities of daily living (ADL) according to Katz index (transferring, eating, continence, dressing, toileting, and bathing).
