# Supplementary material for: The effectiveness of postacute intensive rehabilitation on severe COVID‐19 patients: A case‐control study
Source: Health Sci Rep. 2023 Aug 18;6(8):e1506. doi: 10.1002/hsr2.1506 (PMC10439337; doi:10.1002/hsr2.1506)
Supplement: Supplementary file 2 — Supporting information. [file HSR2-6-e1506-s002.docx]

**STROBE Statement—Checklist of items that should be included in reports of *case-control studies***

**The effectiveness of post-acute intensive rehabilitation on severe COVID-19 patients: a case-control study examining clinical and functional outcomes**

| **Item** | **No** | **Recommendation** |  | |  |
| --- | --- | --- | --- | --- | --- |
| **Title and abstract** | | 1 | (*a*) Indicate the study’s design with a commonly used term in the title or the abstract | | Page 1 |
|  | |  | (*b*) Provide in the abstract an informative and balanced summary of what was done and what was found | | N/A (research letter) |
| **Introduction** | |  |  | |  |
| Background/rationale | | 2 | Explain the scientific background and rationale for the investigation being reported | | Page 2 |
| Objectives | | 3 | State specific objectives, including any prespecified hypotheses | | Page 2 |
| **Methods** | |  |  | |  |
| Study design | | 4 | Present key elements of study design early in the paper | | Page 2 |
| Setting | | 5 | Describe the setting, locations, and relevant dates, including periods of recruitment,  exposure, follow-up, and data collection | | Pages 2-3 |
| Participants | | 6 | (*a*) Give the eligibility criteria, and the sources and methods of case ascertainment and control selection. Give the rationale for the choice of cases and controls | | Page 3  Supplementary Figure S1 |
|  | |  | (*b*) For matched studies, give matching criteria and the number of controls per case | | Pages 3-4 |
| Variables | | 7 | Clearly define all outcomes, exposures, predictors, potential confounders, and effect modifiers. Give diagnostic criteria, if applicable | | Page 3 |
| Data sources/ measurement | | 8* | For each variable of interest, give sources of data and details of methods of assessment (measurement). Describe comparability of assessment methods if there is more than one group | | Page 3 |
| Bias | | 9 | Describe any efforts to address potential sources of bias | | Pages 3-4 |
| Study size | | 10 | Explain how the study size was arrived at | |  |
| Quantitative variables | | 11 | Explain how quantitative variables were handled in the analyses. If applicable,  describe which groupings were chosen and why | | Pages 3-4 |
| Statistical methods | | 12 | (*a*) Describe all statistical methods, including those used to control for confounding | | Pages 3-4 |
|  | |  | (*b*) Describe any methods used to examine subgroups and interactions | | N/A |
|  | |  | (*c*) Explain how missing data were addressed | | Page 3 |
|  | |  | (*d*) If applicable, explain how matching of cases and controls was addressed | | Page 3  Supplementary Figure S1 |
|  | |  | (*e*) Describe any sensitivity analyses | | N/A |
| **Results** | |  |  | |  |
| Participants | | 13* | (a) Report numbers of individuals at each stage of study—eg numbers potentially eligible, examined for eligibility, confirmed eligible, included in the study, completing follow-up, and analysed. | | Supplementary Figure S1 |
|  | |  | (b) Give reasons for non-participation at each stage | | N/A |
|  | |  | (c) Consider use of a flow diagram | | Supplementary Figure S1 |
| Descriptive data | | 14* | (a) Give characteristics of study participants (eg demographic, clinical, social) and  information on exposures and potential confounders | | Page 4  Table 1 |
|  | |  | (b) Indicate number of participants with missing data for each variable of interest | | Table 1 |
| Outcome data | | 15* | Report numbers in each exposure category, or summary measures of exposure | | Table 1 |
| Main results | | 16 | (*a*) Give unadjusted estimates and, if applicable, confounder-adjusted estimates and their precision (eg, 95% confidence interval). Make clear which confounders were adjusted for and why they were included | | Pages 4-5  Table 1  Figure 1 |
|  | |  | (*b*) Report category boundaries when continuous variables were categorized | | Table 1  Figure 1 |
|  | |  | (*c*) If relevant, consider translating estimates of relative risk into absolute risk for a meaningful time period | | Pages 4-5  Table 1  Figure 1 |
| Other analyses | | 17 | Report other analyses done—eg analyses of subgroups and interactions, and sensitivity analyses | | N/A |
| Discussion | |  |  | |  |
| Key results | | 18 | Summarise key results with reference to study objectives | | Page 5 |
| Limitations | | 19 | Discuss limitations of the study, taking into account sources of potential bias or imprecision.  Discuss both direction and magnitude of any potential bias. | | Page 5  Page 5 |
| Interpretation | | 20 | Give a cautious overall interpretation of results considering objectives, limitations, multiplicity of analyses, results from similar studies, and other relevant evidence | | Pages 5-6 |
| Generalisability | | 21 | Discuss the generalisability (external validity) of the study results | | Page 5 |
| **Other information** | |  |  | |  |
| Funding | | 22 | Give the source of funding and the role of the funders for the present study and, if applicable, for the original study on which the present article is based | | Page 6 |
